# Genome Variation in Alcohol Use Disorder by Whole‐Exome Sequencing

**DOI:** 10.1111/adb.70070

**Published:** 2025-07-29

**Authors:** Lei Liu, Bo Zhang, Yong Dong, Li Ping Liu, Jing Ying Wang, Jun Liu, Guang Yu Zhou, Chuan Yi Kang, Xiaorui Hu, Chang Cheng, Na Zhao, Jia Lu, Huaizhi Wang, Jian Hu, Xiaohong Wang

**Affiliations:** ^1^ Department of Psychiatry The First Affiliated Hospital of Harbin Medical University Harbin Heilongjiang China; ^2^ Department of Psychology Shandong Provincial Hospital Affiliated to Shandong First Medical University Jinan Shandong China; ^3^ Department of Psychiatry Changchun Sixth Hospital Changchun China; ^4^ Department of Psychiatry The First Special Hospital of Harbin Harbin Heilongjiang China; ^5^ The Third Hospital of Heilongjiang Province Harbin Heilongjiang China; ^6^ Shenyang Mental Health Center Shenyang Liaoning China

**Keywords:** alcohol use disorder, protein function prediction, rare variants, variants, whole‐exome sequencing

## Abstract

Alcohol use disorder is closely related to genetic and environmental factors. However, the contribution of coding variation to alcohol use disorder susceptibility remains poorly understood. We aimed to identify genetic mutations in alcohol use disorder by whole exon sequencing. We performed whole‐exome sequencing in 83 patients with alcohol use disorder and compared it with exome sequences of healthy controls that were collected from the 1000 Genomes Project. GO and KEGG enrichment analysis and protein interaction analysis were performed for the mutated genes in each group. Three online protein function prediction sites were used to predict whether SNPs/InDels cause protein coding changes. Further, we conducted a rare variant exploration. We identified 106 525 SNV and 19 826 InDel gene mutations in alcohol use disorder. In the healthy and alcohol use disorder groups, mutations in CNTNAP3, ZNF683, ALDPH2, CCHCR1, ZNF45 and ESRRA loci were found to be deleterious mutations in all three sites; CNTNAP3, ZNF683, ALDPH2, CCHCR1, ZNF45 and ESRRA may be potential targets for future precision treatment of alcohol use disorders, and further provide new ideas for drug development.

## Graphical abstract

Whole‐exome sequencing of 83 alcohol use disorder patients identified 106,525 SNVs and 19,826 InDels, revealing six genes (CNTNAP3, ZNF683, ALDH2, CCHCR1, ZNF45, ESRRA) with deleterious mutations through comprehensive bioinformatics analysis. These novel genetic variants represent potential targets for precision medicine in alcohol use disorder treatment. This study bridges knowledge gaps in AUD genetics and provides new insights for personalised therapeutic strategies and drug development.

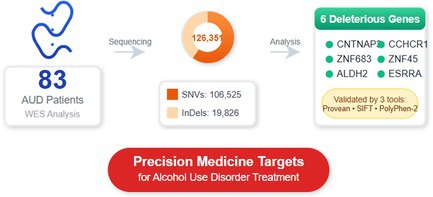

**Abbreviations**
AUD: alcohol use disorder
DSM‐V: Diagnostic and Statistical Manual of Mental Disorders, Fifth Edition
GO: gene ontology
GWAS: genome‐wide association studies
InDel: insertion/deletion mutation
KEGG: Kyoto Encyclopedia of Genes and Genomes
SNV: single nucleotide variant
WES: whole‐exome sequencing

## 1 Introduction

Alcohol use disorder (AUD) causes a huge burden of disease. However, treatments have limited efficacy. Several studies have shown that abnormal gene expression and polymorphism are strongly associated with AUD [1, 2, 3, 4, 5]. The heritability of AUDs is estimated at approximately 50%–60% of the total phenotypic variability [1]. Over the past few decades, extensive research has focused on identifying genetic factors associated with AUD, providing valuable insights into the molecular basis of the disorder.

Genome‐wide association studies (GWAS) have been instrumental in identifying common genetic variants linked to AUD, implicating genes involved in neurotransmission, alcohol metabolism and neurodevelopment. Notably, variations in genes such as *GABRA2*, *ADH1B*, *ALDH2* and *OPRM1* have been associated with altered alcohol consumption behaviours and dependence [6, 7, 8, 9, 10, 11]. However, despite these advancements, a significant proportion of the genetic contribution to AUD remains unexplained. One limitation of GWAS is its focus on common variants with small effect sizes, often overlooking rare coding variants that may have substantial functional consequences [12, 13].

The human exome is 1% to 2% of the entire genome [14, 15]. It is now estimated that more than 85% of diseases associated with DNA variants are derived from exon regions of DNA [16]. So, it is more cost‐effective to use whole‐exome sequencing (WES) to predict DNA changes that may alter protein function [17]. WES is used to identify variations in all coding regions or exons of known genes [18]. WES enables the detection of novel genetic variations that might be missed in traditional GWAS. This method has been successfully applied to various neuropsychiatric disorders [19, 20, 21, 22]. Hill et al. sequenced the exome of multicase families in AUD using a case–control study approach, found six genes that were extremely rare, and identified eight genes with a MAF of 0.001: *ZNF514*, *OXGR1*, *DIEXF*, *TMX4*, *MTBP*, *PON2*, *CRHBP* and *ANKRD46*, along with three protein truncation variants associated with loss of function: AGTRAP, ANKRD46 and PPA1 [23]. Using saliva samples, AshaRani et al. conducted WES on a trio of substance‐use disorder patients (the subject and two family members), which revealed that each person had several genetic variants and most had damaging protein mutations [24].

In this study, by integrating bioinformatics approaches, including gene enrichment and protein interaction analyses, we aimed to identify novel pathogenic mutations and pathways that could serve as potential targets for precision medicine. Our findings will contribute to bridging existing knowledge gaps in AUD genetics and provide new insights into personalized treatment strategies for the disorder.

## 2 Methods

### 2.1 Study Participants

A cohort of 83 patients with AUD was recruited from the First Affiliated Hospital of Harbin Medical University. Blood samples from these patients were collected for WES, as well as simultaneous collection of clinical sample information. All patients were diagnosed with DSM‐V and provided written informed consent, ages 18–60 years old. Exclusion criteria: (1) serious cardiovascular system diseases, nervous system diseases and other major physical diseases that may affect the experimental results; (2) exclusion of substance use disorders other than alcohol, tobacco, and morphine; (3) exclusion of Alzheimer's disease, bipolar disorder and other major mental disorders. The study was authorized and approved by the Harbin Medical University Ethics Committee.

### 2.2 Exome Sequencing

**FIGURE 1:**
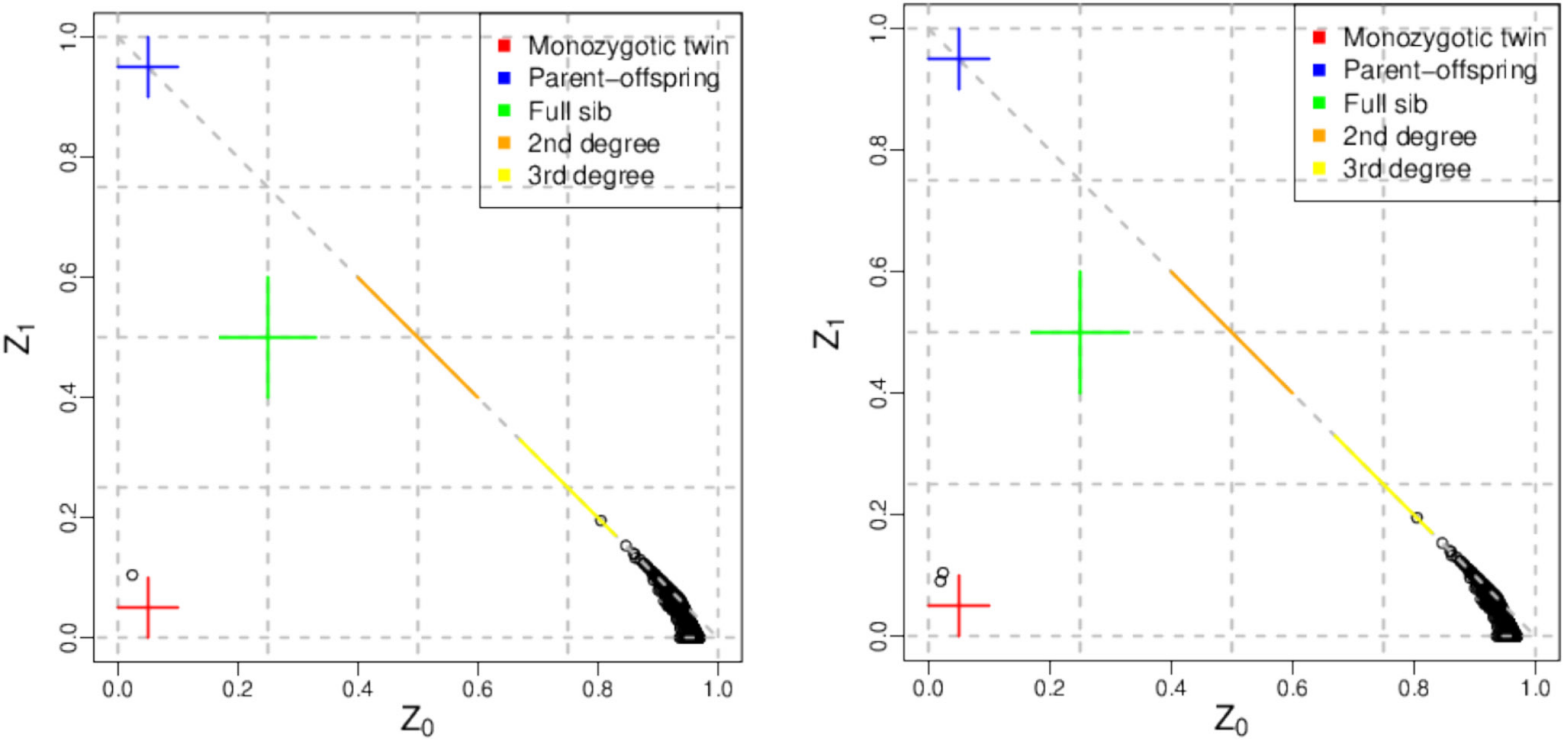
Identity by descent (IBD) kinship chart.

Qualified genomic DNA samples are randomly interrupted by an ultrasonic high‐performance sample processing system (Covaris); after fragment selection, a fragment of about 150–250 bp was obtained. DNA fragment end repair was performed, followed by linear amplification (LMPCR) to prepare a hybridization library. Amplification was performed after capture enrichment with exon microarrays. High‐throughput sequencing was performed on each qualified library using the BGI DNBSEQ platform. We performed WES on 100 patients. The raw sequencing data were filtered, compared and analysed. The average sequencing depth of the target region was approximately 97.52 X. We used the thousand genome data as a health control, downloaded the corresponding vcf files (ftp://ftp.1000genomes.ebi.ac.uk/vol1/ftp/data_collections/1000_genomes_project/release/20190312_biallelic_SNV_and_INDEL/) and then took out 211 of the Chinese samples for Hardy–Weinberg equilibrium testing, genome‐wide, 44 456 loci do not satisfy Hardy–Weinberg equilibrium, and these loci are removed from the control as well as case samples. Then, 211 Chinese samples were tested for IBD to determine the relatedness between samples, which required the removal of sample NA18527, leaving 210 Chinese samples as control samples (Figure 1). The filtering indexes include comparison rate, depth, coverage and chain balance. We used the HaplotypeCaller of GATK v4.1.4 to detect genomic variants, including SNPs and InDel. Next, we used SnpEff (http://snpeff.sourceforge.net/SnpEff_manual.html) software to annotate the result of the variation and the expected effect. The final variation and annotation results will be used for advanced downstream analysis.

### 2.3 Rare Variants

Studies have shown that rare variants that occur at low frequencies in the population and cannot be detected by SNP chips can play an important role in complex diseases, and we also use the SKAT‐O method to study rare variants (MAF < 0.05**)**.

## 3 Results

### 3.1 Single Nucleotide Variant and Insertion/Deletion Identification

**FIGURE 2:**
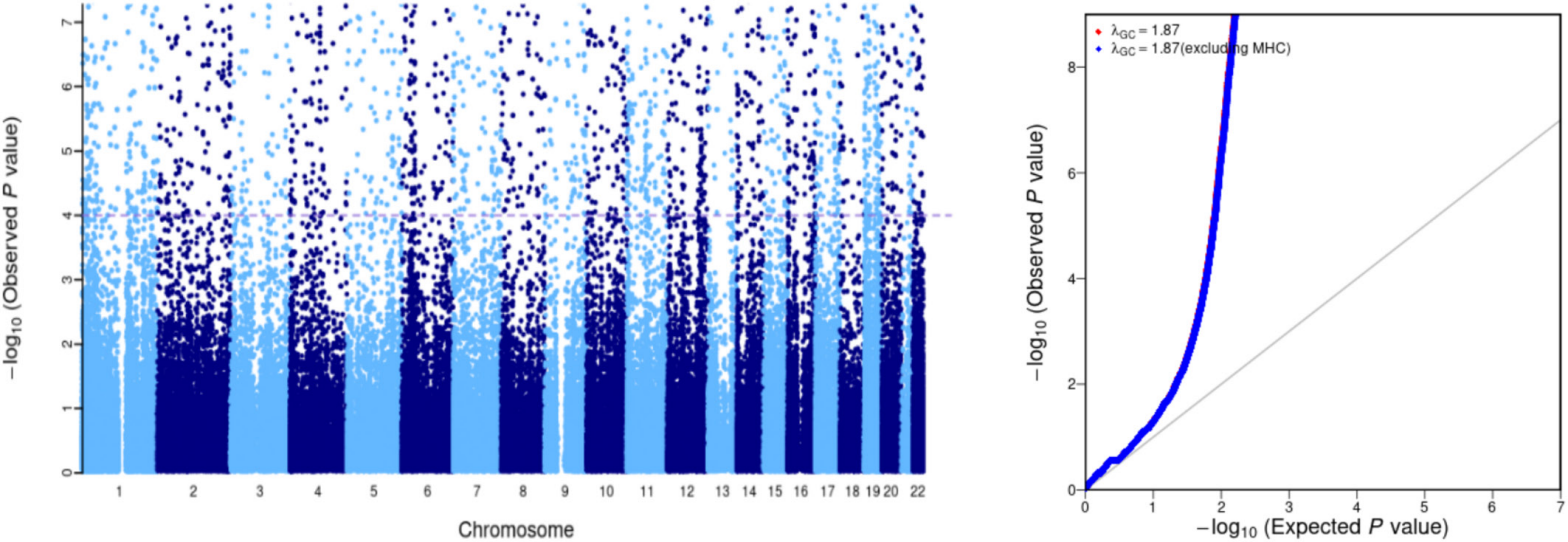
Manhattan plot and QQ plot of common variant associations between alcohol use disorder versus healthy control.

There were 106 525 single nucleotide variants (SNVs) identified in 83 sequenced alcohol‐dependent individuals, 97.99% in the dbSNP database and 92.92% in the 1000 Genomes Project database The Manhattan plot and the QQ plot are shown in Figure 2. There were 2117 newly identified SNPs. Among the overall SNPs, there were 10 332 synonymous mutations and 10 239 missense mutations in the coding region; 34 SNPs changed the stop codon to a nonstop codon, 128 SNPs changed the codon to a stop codon, 40 SNPs changed the start codon to a nonstart codon, and 143 SNPs changed the splice acceptor or splice donor in the splice site region.

There were 19 826 InDel found in all samples, 79.14% in the dbSNP database and 50.17% in the 1000 Genomes Project database. There were 4073 newly discovered InDel. Among the total number of InDel, there were 271 shift mutations in the coding region: 0 InDel changed the stop codon to a nonstop codon, 3 InDel changed the start codon to a nonstart codon and 50 InDel changed the splice acceptor or splice donor in the splice site region.

Based on the *p* value, the top 10 genes with rare variants in each group are listed (ascending order) (Tables 1 and 2).

**TABLE 1:** Results of singlebase analysis for each group (MAF > 0.05).

Group	RS	Pos	Minor/major	Fre_minor_case	Fre_minor_control	OR (fisher)_L95_U95	Pval (fisher)	Gene	Functionype
Alcohol use disorder vs. healthy control	chr11	11:130 418 902	ACCT/A	1	0	0.00 [0.00,0.00]	3.33E‐148	ADAMTS8	Intronic
	chr5	5:73 128 688	AC/A	1	0	0.00 [0.00,0.00]	3.33E‐148	TMEM171	Intronic
	chr1	1:59 762 638	TTC/T	0.99	0	0.00 [0.00,0.00]	3.79E‐146	FGGY	UTR3
	chr4	4:6 372 503	CA/C	0.99	0	0.00 [0.00,0.00]	3.79E‐146	PPP2R2C	Intronic
	chr2	2:215 379 458	A/AGTAC	0.99	0	0.00 [0.00,0.00]	7.99E‐144	FN1	Intronic
	chr11	11:1 193 310	GC/G	0.99	0	0.00 [0.00,0.00]	1.04E‐142	MUC5AC	Intronic
	chr19	19:18 433 867	GC/G	0.99	0	0.00 [0.00,0.00]	1.96E‐142	SSBP4	Intronic
	chr1	1:92 247 041	G/GAA	0.98	0	0.00 [0.00,0.00]	1.13E‐141	GLMN	Intronic
	chr3	3:52 471 742	GAT/G	0.98	0	0.00 [0.00,0.00]	1.20E‐139	NISCH	Intronic
	chr20	20:62 661 021	GAGCCCCC/G	0.98	0	0.00 [0.00,0.00]	1.69E‐136	SLCO4A1	Intronic
	chr1	1:1 707 744	G/A	0.5	0	0.00 [0.00,0.00]	2.08E‐05	CDK11A	Intronic

**TABLE 2:** Results of genebase analysis for each group (MAF < 0.05).

Group	Chrom	Skat_p	Frac_with_rare	Gene	Category
Alcohol use disorder vs. health control group	chr12	NA	NA	AKAP3	5.disruptive
	chr19	NA	NA	MADCAM1	2.deleterious_polyphen
	chr5	NA	NA	ZDHHC11B	2.deleterious_polyphen
	chr13	NA	NA	OXGR1	5.disruptive
	chr2	NA	NA	RETSAT	4.deleterious_strict
	chr11	NA	NA	OR52D1	6.frameshift
	chr17	NA	NA	FOXK2	2.deleterious_polyphen
	chr2	NA	NA	MOGAT1	6.frameshift
	chr21	NA	NA	BAGE;BAGE4;BAGE5	2.deleterious_polyphen
	chr19	NA	NA	FIZ1	1.non‐synonymous

### 3.2 GO and KEGG Pathway Enrichment Analysis

#### 3.2.1 Singlebase Correlation Analysis

**FIGURE 3:**
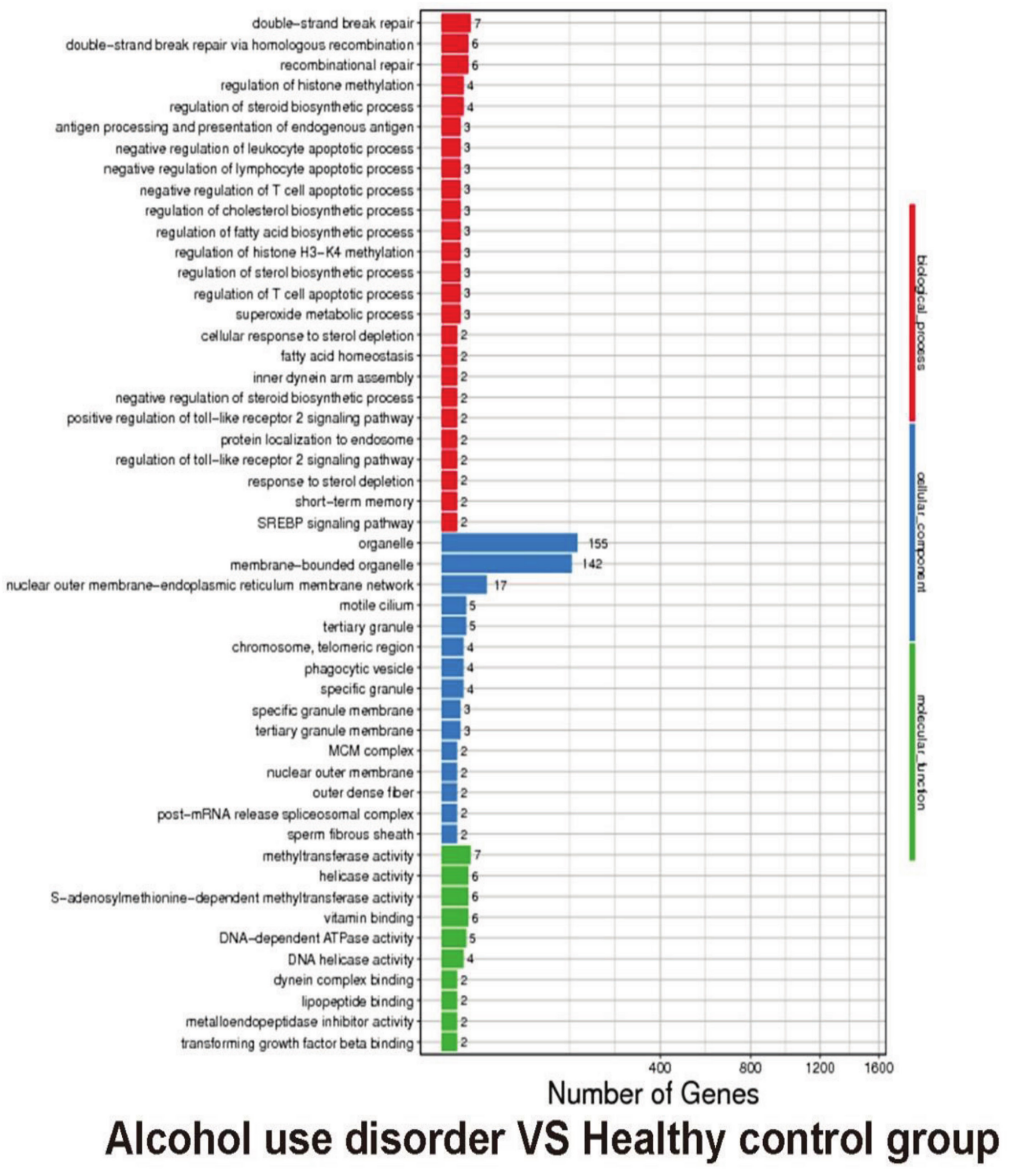
Biological function enrichment analysis of singlebase in alcohol use disorder versus health control group.

Results of GO analysis in singlebase showed that AUD vs. health control group: BP: response to stimulus; multicellular organismal process; localization; CC: cytoplasm, cell periphery, plasma membrane; MF: anion binding. small molecule binding, carbohydrate derivative binding (Figure 3).

**FIGURE 4:**
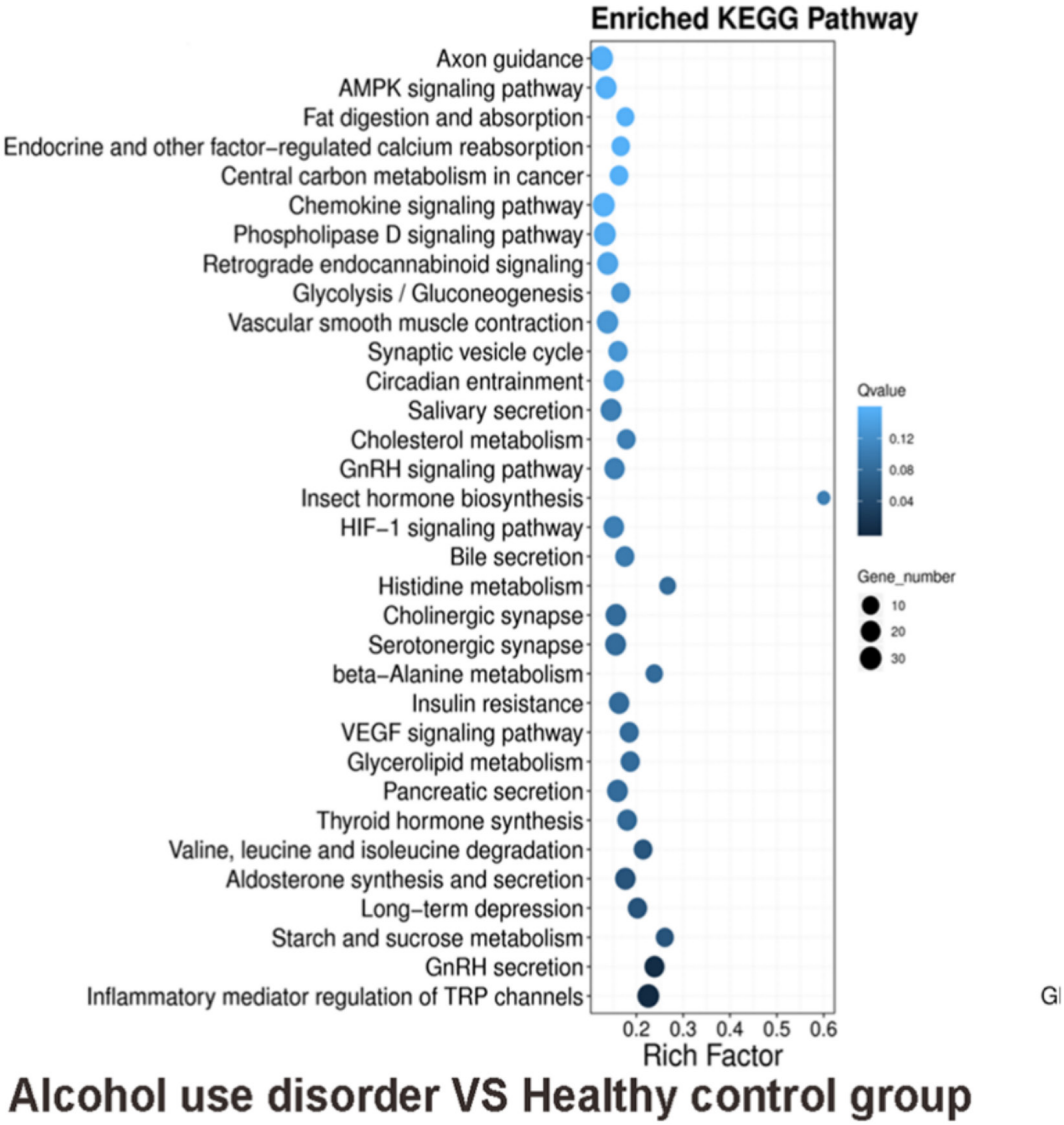
KEGG enrichment analysis of singlebase in alcohol use disorder versus healthy control group.

Results of KEGG analysis in singlebase showed that AUD versus health control group: inflammatory mediator regulation of TRP channels, GnRH secretion, VEGF signalling pathway, insulin resistance, HIF‐1 signalling pathway, GnRH signalling pathway, cholesterol metabolism, phospholipase D signalling pathway, axon guidance, hedgehog signalling pathway (Figure 4).

#### 3.2.2 Gene‐Based Correlation Analysis

**FIGURE 5:**
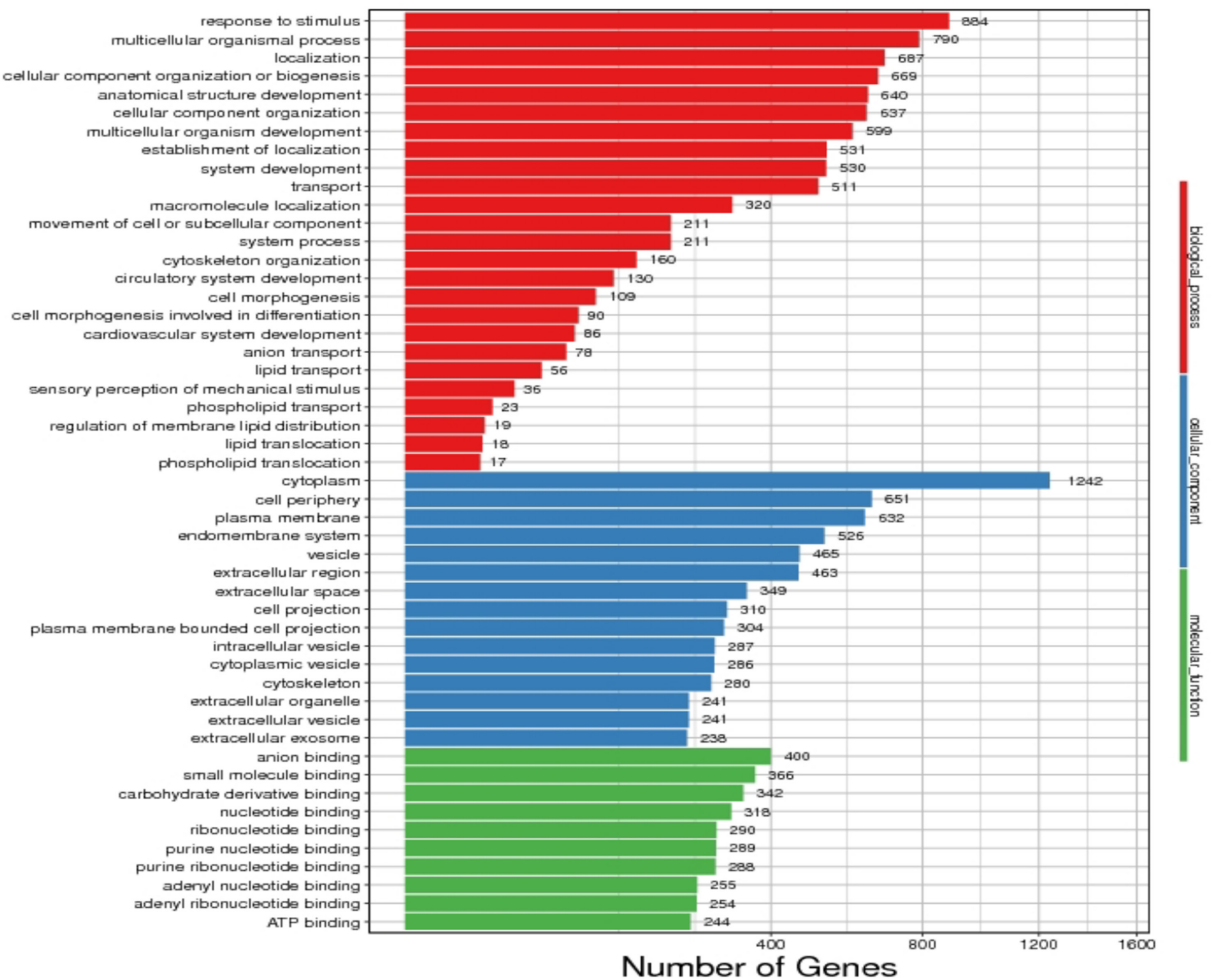
Biological function enrichment analysis of genebase in alcohol use disorder versus health control group.

We conducted GO and KEGG pathway analysis of the mutant genes; results of GO analysis in genebase showed that AUD versus health control group: BP: double−strand break repair; double−strand break repair via homologous recombination; recombinational repair; CC: organelle, membrane−bounded organelle, nuclear outer membrane−endoplasmic reticulum membrane network; MF: methyltransferase activity, helicase activity, S–adenosylmethionine−dependent methyltransferase activity (Figure 5).

**FIGURE 6:**
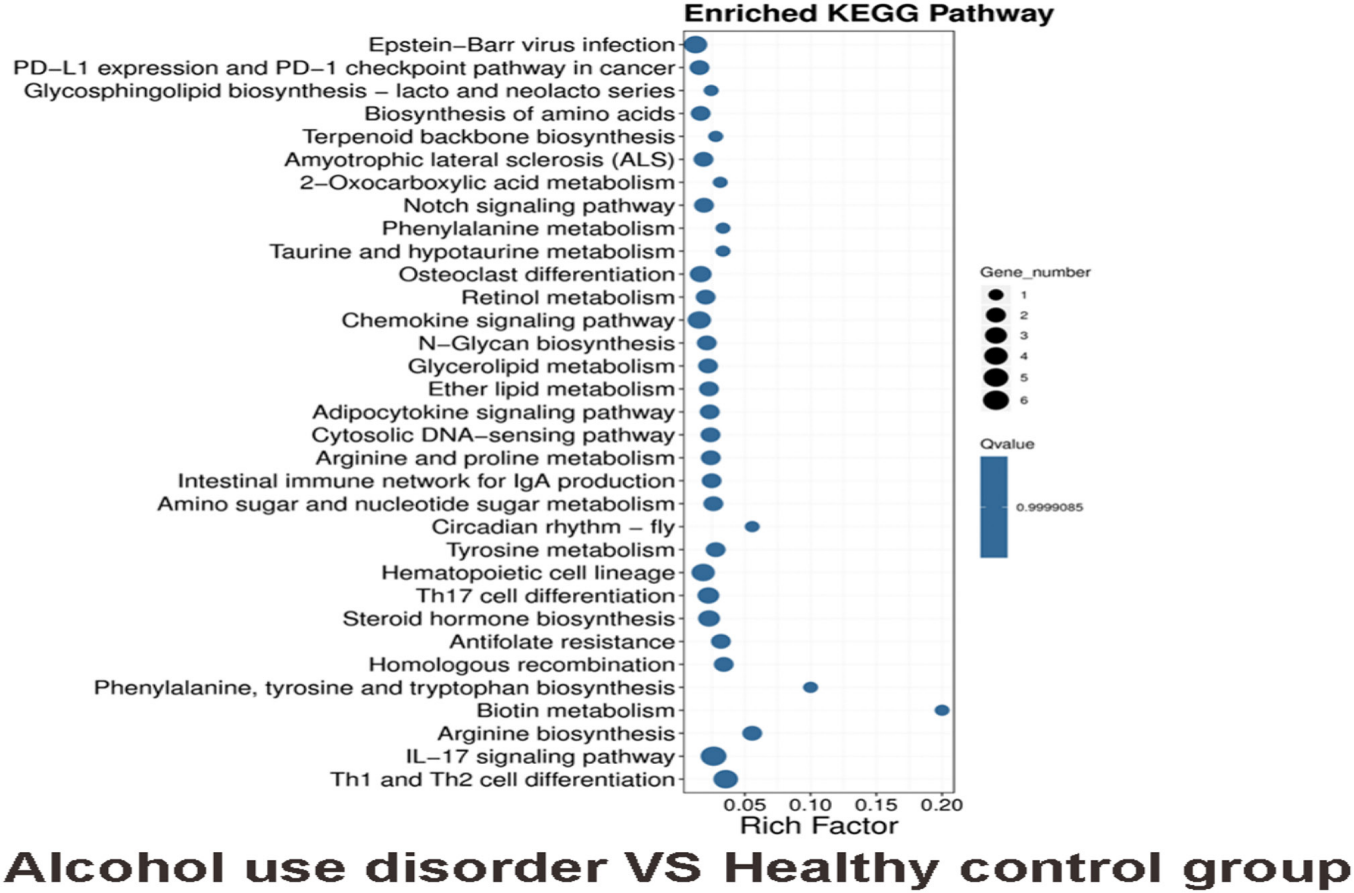
KEGG enrichment analysis of genebase in alcohol use disorder versus healthy control group.

Results of KEGG pathway analysis in genebase showed that AUD versus health control group: Th1 and Th2 cell differentiation, IL‐17 signalling pathway, arginine biosynthesis, biotin metabolism (Figure 6);

### 3.3 PPI

**FIGURE 7:**
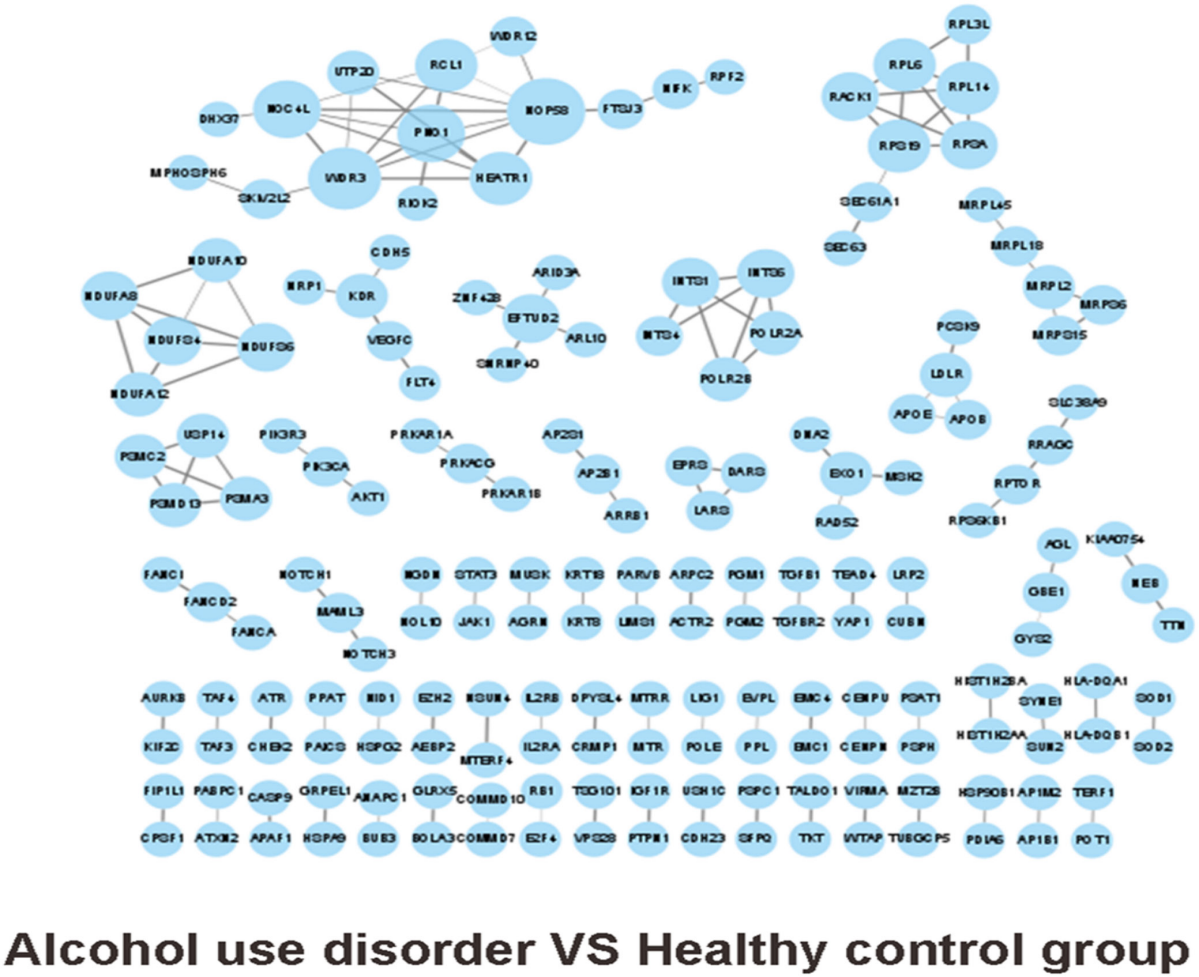
PPI network of singlebase in alcohol use disorder versus healthy control group.

**FIGURE 8:**
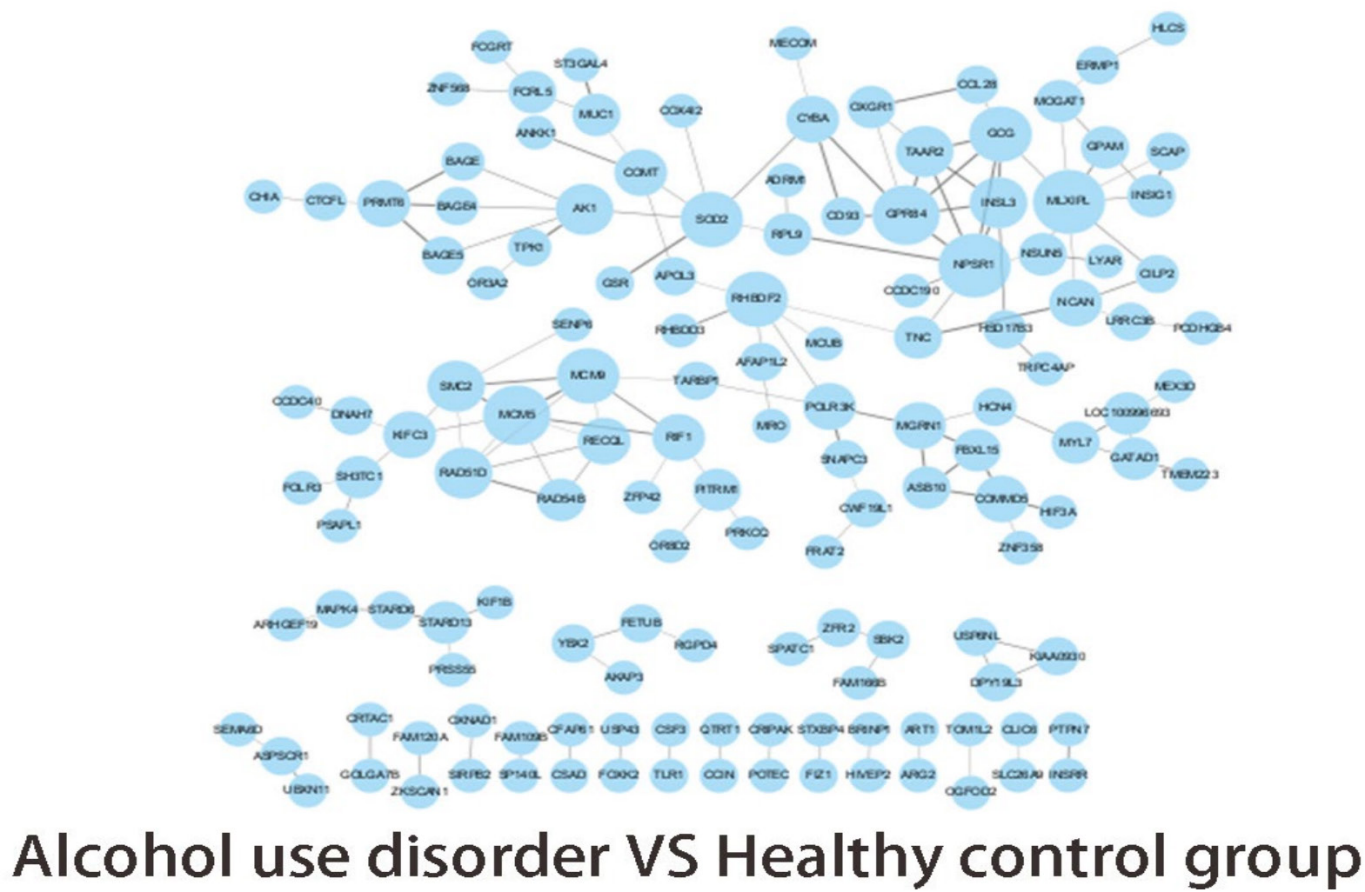
PPI network of genebase in alcohol use disorder versus healthy control group.

PPI was applied to analyse the interactions of four groups of mutant genes. We selected the top 150 gene interactions in descending order of the interaction score and used Cytoscape for the graphs. PPI based on association analysis of single base (Figure 7); gene‐based association analysis of PPI (Figure 8).

### 3.4 Pathogenicity Analysis

We used three online sites (Proven. Sift. Polyphen‐2) to predict protein conformational changes and thus assess whether this mutation site is a deleterious mutation (Tables 3, 4, 5, 6).

**TABLE 3:** Harmful mutations screened in the Health and Disease group using the Sift online site.

Gene	Functionype	SIFT_score	SIFT_pred
*ZFP2*	Missense	0.02	D
*PSPH*	Missense	0	D
*CNTNAP3*	Missense	0.02	D
*ZNF683*	Missense	0.04	D
*CFHR3*	Intronic	0	D
*ARF3*	UTR3	0	D
*OR4L1*	Missense	0	D
*OR4L1*	Missense	0.01	D
*ALDH2*	Missense	0	D
*LOC100130698*	ncRNA_exonic	0.02	D
*REPIN1*	Missense	0	D
*HELZ2*	Missense	0.02	D
*MT1A*	Missense	0.01	D
*RAB36*	UTR3	0	D
*D2HGDH*	Intronic	0	D
*LINC00905*	ncRNA_exonic	0	D
*MYOM2*	Missense	0.04	D
*FAM201A*	ncRNA_exonic	0	D
*CCHCR1*	Missense	0	D
*MYH9*	Missense	0.04	D
*KRT87P;KRT86*	Intergenic	0	D
*GALNS*	Missense	0.01	D
*ZNF45*	Missense	0.01	D
*MRGPRX2*	Missense	0.02	D
*LOC728975*	ncRNA_exonic	0	D
*LINC00905*	ncRNA_exonic	0	D
*KDM2B*	Intronic	0.05	D
*PSMG4*	Missense	0	D
*FGF18;SMIM23*	Intergenic	0	D
*MINDY4*	Missense	0	D
*LRRK1*	Intronic	0	D
*BRWD1‐IT2*	ncRNA_exonic	0	D
*LOC100996419*	ncRNA_exonic	0	D
*CMYA5*	Missense	0	D
*EZH2*	Missense	0.01	D
*ESRRA*	Missense	0	D
*HLA‐DQB1*	Missense	0.01	D
*MMP27*	Missense	0.02	D

*Note:* SIFT‐score ranges from 0 to 1:0–0.05: mutation sites are considered to be deleterious, which can lead to changes in protein function, and the smaller the value, the greater the possibility of causing changes in protein function; 0.05–1: It is believed that the mutation site is benign and has little or no impact on protein function if it is Tolerated, and the more the value is close to 1, the less harmful it will be to protein function.

**TABLE 4:** Harmful mutations screened in the Health and Disease group using the Polyphen2_HDIV online sit.

Gene	Functionype	Polyphen2_HDIV_score	Polyphen2_HDIV_pred
TAF4	Missense	0.637	P
CNTNAP3	Missense	1	D
ZNF683	Missense	1	D
CFHR3	Intronic	0.855	P
FAM198A	Missense	0.65	P
KNDC1	Missense	0.867	P
COLEC12	Missense	1	D
OBP2A	Missense	0.994	D
OR4L1	Missense	0.929	P
OR4L1	Missense	0.976	D
ALDH2	Missense	1	D
REPIN1	Missense	0.645	P
MT1A	Missense	0.928	P
FAM173B	Missense	0.996	D
MYOM2	Missense	0.999	D
ALPK2	Missense	0.994	D
FAM201A	ncRNA_exonic	0.997	D
CCHCR1	Missense	1	D
CCHCR1	Missense	1	D
SPAG11A	Missense	0.994	D
ZNF45	Missense	1	D
MRGPRX2	Missense	0.976	D
LOC728975	ncRNA_exonic	0.711	P
CR1	Missense	0.995	D
CR1	Missense	0.643	P
CMYA5	Missense	0.91	P
EZH2	Missense	0.898	P
ESRRA	Missense	1	D
AHNAK2	Missense	0.975	D
LAMA4	Missense	1	D
MUC17	Missense	0.971	D

*Note:* Polyphen2_HDIV_score is used to predict the potential effects of non‐synonymous mutations (i.e., amino acid substitution) on protein structure and function. According to the specific value of Polyphen2_HDIV_score, the prediction results can be divided into the following categories: D: probably damaging: when Polyphen2_HDIV_score ≥ 0.957, the mutation is predicted to be harmful, indicating that the mutation is likely to have a negative impact on protein function; P: possibly damaging: when 0.453 ≤ Polyphen2_HDIV_score < 0.957, it is predicted that the mutation may be harmful, but the degree of uncertainty is high; benign (B: benign): when Polyphen2_HDIV_score < 0.453, the mutation is predicted to be benign, indicating that the mutation has little effect on protein function.

**TABLE 5:** Harmful mutations screened in the Health and Disease group using the Polyphen2_HVAR online sit.

Gene	Functionype	Polyphen2_HVAR_score	Polyphen2_HVAR_pred
*CNTNAP3*	Missense	0.994	D
*ZNF683*	Missense	0.996	D
*CFHR3*	Intronic	0.681	P
*COLEC12*	Missense	0.998	D
*OBP2A*	Missense	0.937	D
*OR4L1*	Missense	0.598	P
*OR4L1*	Missense	0.608	P
*ALDH2*	Missense	0.98	D
*REPIN1*	Missense	0.533	P
*MT1A*	Missense	0.642	P
*FAM173B*	Missense	0.851	P
*MYOM2*	Missense	0.967	D
*ALPK2*	Missense	0.906	P
*FAM201A*	ncRNA_exonic	0.969	D
*CCHCR1*	Missense	0.966	D
*CCHCR1*	Missense	1	D
*SPAG11A*	Missense	0.993	D
*ZNF45*	Missense	0.999	D
*MRGPRX2*	Missense	0.817	P
*CR1*	Missense	0.984	D
*CMYA5*	Missense	0.469	P
*EZH2*	Missense	0.603	P
*ESRRA*	Missense	1	D
*LAMA4*	Missense	0.994	D

*Note:* PolyPhen2_HVAR_score is used to predict the effect of non‐synonymous mutations (amino acid substitution) on protein structure and function. The score ranges from 0 to 1, with the higher the value, the greater the mutation's potential impact on protein structure and function. Rating range and predicted results. 0 to 0.446: The prediction is benign, indicating that the mutation has little effect on protein function; 0.447 to 0.908: The prediction is ‘possibly damaging’, indicating that the mutation has a certain probability of negatively affecting protein function; 0.909 to 1: A prediction of ‘probably damaging’ indicates that the mutation has a high probability of negatively affecting protein function.

**TABLE 6:** Harmful mutations screened in the Health and Disease group using the PROVEAN online sit.

Gene	Functionype	PROVEAN_score	PROVEAN_pred
*ZFP2*	Missense	−6.84	D
*ZNF83*	Missense	−2.57	D
*PSPH*	Missense	−3.1	D
*CNTNAP3*	Missense	−8.1	D
*ZNF683*	Missense	−2.57	D
*CFHR3*	Intronic	−6.7	D
*FAM198A*	Missense	−2.51	D
*OR4L1*	Missense	−4.39	D
*OR4L1*	Missense	−5.57	D
*ALDH2*	Missense	−3.42	D
*SPRR1B*	Missense	−2.52	D
*REPIN1*	Missense	−2.57	D
*HELZ2*	Missense	−3.75	D
*MT1A*	Missense	−4.2	D
*FAM173B*	Missense	−2.73	D
*ALPK2*	Missense	−3.72	D
*CCHCR1*	Missense	−3.32	D
*CCHCR1*	Missense	−3.03	D
*ZNF45*	Missense	−7.47	D
*MRGPRX2*	Missense	−4.62	D
*PABPC1*	Missense	−3.51	D
*PABPC1*	Missense	−4.69	D
*CMYA5*	Missense	−4.29	D
*ESRRA*	Missense	−6.3	D
*AHNAK2*	Missense	−2.87	D
*LAMA4*	Missense	−3.37	D

*Note:* PROVEAN_score (protein variation effect analyzer score) is an indicator used to evaluate the effect of protein variation on protein function. Generally, variants with a PROVEAN_score of less than −2.5 are considered to have a higher likelihood of having an effect on protein structure or function.

In the healthy versus alcohol‐dependent group, the genes jointly identified as deleterious mutations by the three online sites were CNTNAP3, ZNF683, ALDH2, CCHCR1, ZNF45, and ESRRA.

## 4 Discussion

We performed WES combined with bioinformatics analysis in 83 patients with AUDs to investigate the potential application of WES in determining the genetic cause of AUDs, which has rarely been studied before. There were 106 525 SNVs and 19 826 InDels identified in 83 sequenced alcohol‐dependent individuals. Using three online sites to identify whether SNPs/InDels cause protein coding changes and amino acid alterations, we found that CNTNAP3, ZNF683, ALDPH2, CCHCR1, ZNF45 and ESRRA were deleterious mutations in the health and AUD groups; the detected mutation ALDH2 has previously been identified to play a role in AUDs. It showed that ALDH2 variation is related to hazardous drinking and facial flushing in Thai males [25]. Northeast Asians are virtually exclusively carriers of ALDH2*2. The allele ALDH2*2 has the strongest correlation with alcoholism. Asians with the ALDH2*2 gene exhibit stronger protective effects [26]. Wang et al. analysed the WES data from the Yale‐Pennsylvania cohort and the UK Biobank, further confirming the variations in ADH1B and ADH1C in AUD. Furthermore, they also discovered the previously unreported gene CNST IFIT5 in rare mutations [27]. The ALDH2*1 allele was found as a risk factor for alcohol abuse and alcohol cirrhosis, and combined genotypes of ADH1B rs1229984, ADH1C rs698 and ALDH2 rs671 with non‐acetaldehyde accumulation increase alcohol cirrhosis risk [28].

The function of CNTNAP3 has not been fully detected. CNTNAP3 was highly expressed in the cortex and hippocampus of the mouse postnatal brain at P7 and P14 [29]. Cntnap3^−/−^ mice exhibited deficits in social behaviour, cognitive tasks and prominent repetitive behaviours [30]. Furthermore, a deletion of 9p12, which contains CNTNAP3, was found in one mental retardation (MR) patient [31]. Some evidence suggests that Caspr3 is involved in motor control and learning. Caspr3 may also contribute to neuronal activity associated with motor learning [32]. At the same time, alcohol is a central nervous system depressant that causes cognitive impairment and motor incoordination through its effects on several neurotransmitters [33]. Taken together, CNTNAP3 may be a candidate gene for alcohol dependence. ZNF683 is key in ZNF683/HOBIT for differentiating the human NK‐cell lineage [34], leading to NK cell cytotoxic activity impairment [35]. Studies have shown that chronic alcohol consumption inhibits NK cell maturation. However, the role of ZNF683 in AUD has not been explored.

As a centrosomal P‐body protein, CCHCR1 (Coiled‐Coil α‐Helical Rod protein 1) may regulate several cytoskeleton‐mediated processes, including cell adhesion and division [36]. CCHCR1 is involved in the pathogenesis of psoriasis [36, 37, 38], skin cancer [39] and alopecia areata [40]. AUD is an independent risk factor for psoriasis [41], and alcohol intake was positively correlated with the severity of psoriasis [42]. CCHCR1 has not been explored in alcohol dependence but may serve as a prospective drug target in the future.

*ZNF45* gene expression was negatively associated with BMI, suggesting that this gene is associated with obesity and metabolic disorders [43]. GWAS of developmental dyslexia (DD) revealed that ZNF45 was expressed lower in schizophrenia patients than in the controls [44]. A whole‐transcriptome splicing association study found that ZNF45 was associated with susceptibility to Alzheimer's disease [45]. However, AUDs increase the risk of metabolic and cognitive impairment [46, 47]. Further work is needed in the future to clarify better the potential link between this gene and AUD. Previous studies have identified ZNF514 as an extremely rare gene in AUD (MAF of 0.001) [23]. Both ZNF514 and ZNF45 belong to the zinc finger protein family, which are classified as transcription factors involved in the regulation of gene expression and are involved in immune responses; the specific mechanism remains to be studied [48].

The oestrogen‐related receptor alpha (ESRRA) is an orphan nuclear receptor (NR) that significantly influences cellular metabolism. ESRRA controls the transcription of metabolic genes, including those about autophagy and mitochondrial turnover, and is primarily expressed in metabolically active tissues. It was demonstrated that when exposed to cold, ERRα null mice have trouble regulating their body temperature in the core [49, 50, 51]. The *ESR1* gene encodes for oestrogen receptor alpha. Certain polymorphisms in the oestrogen receptor alpha (ESR1) have been linked to a decreased grey matter volume in the cerebral and cerebellar cortex as well as an increased risk of cognitive impairment in older women [52, 53]. The ESR1 SNPs rs6902771, rs11155819, rs6557171, rs2982683 and rs2982712 were shown to be jointly associated with alcohol dependency in the genetic subproject. The involvement of the rs6902771 single nucleotide polymorphism (SNP) of the oestrogen receptor 1 gene (ESR1) in male alcohol dependence was discovered through a genome‐wide case–control analysis and validated in a subsequent study [54].

Overall, through the prediction of variation and rare variation, we found new candidate genes, indicating the direction for future AUD research. A major limitation of our study is the lack of internal health controls and the need for further Sanger validation. Another disadvantage is the small sample size. In addition, both in vivo and in vitro experiments are needed to validate the new genes identified. Future studies will be necessary to confirm our findings and extend our current results.

## 5 Conclusion

This study could serve as a model for future personalized interventions based on precision medicine to identify potential disease‐causing genetic variants. At the same time, it provides new ideas for precise therapy and drug development of AUD.

## Author Contributions

Lei Liu designed the study. Bo Zhang wrote the manuscript. Yong Dong, Li Ping Liu, Jing Ying Wang, Jun Liu, Guang Yu Zhou, Chuan Yi Kang, Xiaohong Wang, Xiaorui Hu, Chang Cheng, Na Zhao, Jia Lu and Huaizhi Wang participated in data collection. Jian Hu revised the manuscript.

## Conflicts of Interest

The authors declare no conflicts of interest.

## Data Availability

The datasets presented in this study can be found in online repositories.
